# High prevalence of quasi-legal psychoactive substance use among male patients in HIV care in Japan: a cross-sectional study

**DOI:** 10.1186/s13011-017-0097-2

**Published:** 2017-02-23

**Authors:** Kanna Hayashi, Chihiro Wakabayashi, Yuzuru Ikushima, Masayoshi Tarui

**Affiliations:** 1 British Columbia Centre for Excellence in HIV/AIDS, St. Paul’s Hospital, 608-1081 Burrard Street, Vancouver, BC V6Z 1Y6 Canada; 2 Faculty of Health Sciences, Simon Fraser University, Blusson Hall, Room 11300, 8888 University Drive, Burnaby, BC V5A 1S6 Canada; 3 Department of Health Sciences, Saitama Prefectural University, 820 Sannomiya, Koshigaya-shi, Saitama 343-8540 Japan; 4 Positive Living and Community Empowerment Tokyo (Place Tokyo), 4-11-5-403 Takadanobaba, Shinjuku-ku, Tokyo, 169-0075 Japan; 5 Faculty of Letters, Keio University, 2-15-45 Mita, Minato-ku, Tokyo, 108-8345 Japan; 6 BC Centre for Excellence in HIV/AIDS, 1026 Nelson Street, Vancouver, BC V6E 4S7 Canada

**Keywords:** New psychoactive substance, HIV/AIDS, Drug and narcotic control, Men who have sex with men, Epidemiology, Japan

## Abstract

**Background:**

Syndemics of illicit drug use and HIV remain as significant public health issues around the world. There has been increasing concern regarding the rapidly growing market of new psychoactive substances, particularly in Asia. In response, the Japanese government has increasingly banned such substances in recent years. We sought to identify the prevalence and correlates of use of quasi-legal psychoactive substances among people living with HIV/AIDS (PLHIV) in Japan.

**Methods:**

Data were derived from a nationwide survey of PLHIV conducted at nine leading HIV/AIDS care hospitals between July and December 2013. The prevalence and correlates of the use of quasi-legal psychoactive substances (e.g., synthetic cannabinoids, cathinone derivatives, etc. that had not been prohibited from using at the time of survey) among male participants were examined using multivariate survey logistic regression.

**Results:**

Among 963 study participants, the majority (95.3%) were male. The most commonly used drug among men was quasi-legal psychoactive substances (55.3% ever and 12.8% in the previous year). In multivariate analysis, the lifetime use of tryptamine-type derivatives (i.e., 5-MeO-DIPT or N,N-diisopropyl-5-methoxytryptamine) (adjusted odds ratio [AOR]: 2.42; 95% confidence interval [CI]: 1.36–4.28) and methamphetamine/amphetamine (AOR: 3.59; 95% CI: 2.13–6.04) were independently associated with recent quasi-legal psychoactive substance use.

**Conclusions:**

In our sample of male PLHIV in Japan, quasi-legal psychoactive substances were the most commonly used drugs. Individuals who had ever used tryptamine-type derivatives or methamphetamine/amphetamine were more likely to report recent quasi-legal psychoactive substance use, suggesting a potential shift in drug use patterns from regulated to unregulated substances among this population. These findings indicate a need for further research to examine implications for HIV care.

## Background

Many countries in the world are contending with syndemics of illicit drug use and HIV/AIDS [1, 2]. In Asia, unsafe injection use of opioids has traditionally been a key driver of HIV epidemics [2]. However, a growing body of research indicates that non-injection use of amphetamine-type stimulants is also common, and associated with an elevated HIV risk [2–4]. Specifically, methamphetamine use has been shown to be highly prevalent, particularly among men who have sex with men (MSM), increase HIV seroconversion due to the associated risky sexual behaviour, and compromise HIV treatment outcomes [5–7]. However, little is known about the extent and patterns of use of other synthetic drugs in this context. This is concerning because the Asia-Pacific region has recently experienced explosive increases in the availability of a variety of quasi-legal, mostly synthetic psychoactive substances [8]. These substances are designed to mimic the pharmacological effects of regulated substances including methamphetamine [8].

In Japan, the prevalence of illicit drug use in the general population has historically been low at <3% (lifetime use) [9]. The prevalence of HIV infection has also been low at <0.1% among the general population over the past three decades [10]. However, as in other parts of Asia, the burden of HIV is disproportionately concentrated among MSM [10]. It has been estimated that MSM accounted for over half of the recorded HIV diagnoses [11]. Previous studies reported a very high prevalence (40%) of illicit drug use among HIV-positive MSM in one hospital in Tokyo [12]. Illicit drug use was identified as an independent predictor of loss to follow-up for HIV care and hepatitis C virus infection in this setting [13, 14]. However, these studies did not fully differentiate the use of traditional illicit drugs (e.g., methamphetamine, heroin) and other quasi-legal psychoactive substances.

In response to the explosive spread of quasi-legal synthetic psychoactive substances [15], the Japanese government has made a series of important changes to the drug control legislation over the past decade. Through the 2006 amendment of the Pharmaceutical Affairs Law, quasi-legal synthetic drugs that were deemed to have the potential to cause health-related harms were newly classified as “Designated Substances” [15]. The supply of these substances for non-medical purposes was prohibited [15]. By March 2013, 106 substances, including synthetic cannabinoids, tryptamine-type derivatives and phenethylamine derivatives, were listed as Designated Substances [15, 16]. Since 2013, generic scheduling for certain types of synthetic cannabinoids and cathinone derivatives has also been introduced [16].

Despite the tighter control of supply, the possession and consumption of Designated Substances have remained largely unregulated. By March 2013, only 13 of the 106 Designated Substances, including mephedrone and tryptamine-type derivatives (e.g., 5-MeO-DIPT and *Foxy* [N,N-diisopropyl-5-methoxytryptamine]), have been categorized as “Narcotics” under the Narcotics and Psychotropics Control Law and thereby have been prohibited from possessing and consuming [16]. As such, many Designated Substances have continued to be sold as herbal products, aroma products and bath salts, even in vending machines on the street.

While drug use patterns could be affected by these legislation changes, there is a paucity of epidemiologic data regarding the use of quasi-legal psychoactive substances (i.e., substances that have not been prohibited from possessing or consuming under the Narcotics and Psychotropics Control Law) in Japan [9]. Given the previous studies suggesting that illicit drug use could compromise HIV care and increase adverse health outcomes among HIV-positive MSM in this setting [13, 14], we sought to identify the prevalence and correlates of quasi-legal psychoactive substance use among a large nationwide sample of people living with HIV/AIDS (PLHIV) in Japan.

## Methods

### Study design

Data for this study were derived from a nationwide cross-sectional survey of PLHIV conducted at nine leading HIV/AIDS referral hospitals in Japan between July and December 2013. In brief, beginning in 2003, this is a quinquennial survey that aims to investigate the health and lifestyles among PLHIV in Japan. The nine hospitals included in the survey represent tertiary care hospitals and collectively cover the eight major geographical regions in Japan. In each hospital, HIV/AIDS medical professionals were asked to recruit their patients living with HIV/AIDS in a serial manner as patients visit their hospitals. They explained the study objectives and procedures to potential participants, and obtained informed consent from them. The recruitment was continued until approximately 40% of their patients provided informed consent. While we did not count the number of patients who refused to participate in the survey, informal reports from hospitals indicated that such cases were rare. Consented patients were asked to self-complete an anonymous questionnaire and return it to an independent researcher using a self-addressed, stamped envelope provided with the questionnaire. The questionnaire elicited a range of information, including socio-demographic characteristics, HIV-related clinical histories, general health status, and substance use histories. All consented individuals received a 500 Japanese Yen (approximately US$5.00) gift certificate. The study was approved by the research ethics boards at the Saitama Prefectural University as well as each participating hospital.

### Participants and measures

In total, 1,100 (61.6%) of 1,786 consented individuals completed and returned the questionnaire, including 51 (4.7%) females. For a gender-based descriptive analysis of drug use patterns, the sample was restricted to 963 (87.5%) individuals (918 males and 45 females) who had complete data. For an analysis examining correlates of past year quasi-legal psychoactive substance use among men, the sample was further restricted to 895 male participants with complete data.

For the present analysis, the primary outcome of interest was reporting quasi-legal psychoactive substance use in the previous year, defined as answering “Yes” to a question: “In the past year, have you used quasi-legal drugs in the form of herbal products, aroma liquid, powder, oil, bath salts, or amyl nitrites including ‘rush’ and ‘poppers’?” As such, quasi-legal psychoactive substances encompassed a range of substances that had been largely regulated as Designated Substances but had not been prohibited from possessing or consuming under the Narcotics and Psychotropics Control Law by December 2013. Specifically, these substances would include synthetic cannabinoids, synthetic cathinones, phenethylamine and thiophene derivatives, and amyl nitrites (e.g., *rush*, *poppers*). The term “quasi-legal drugs” was used because it commonly referred to the aforementioned set of quasi-legal psychoactive substances of our interest in Japan, and also because people usually do not know the exact substance contained in the product.

Then, we selected a range of explanatory variables that we hypothesized were potentially associated with the outcome. Socio-demographic characteristics included: age; education (< secondary education vs. ≥ secondary education); currently living alone (yes vs. no) and annual income (<1 million, 1–2.99 million, 3–4.99 million vs. ≥ 5 million Yen). HIV-related measures included: antiretroviral therapy (ART) use (currently on ART vs. never on ART); ever had AIDS (yes vs. no); perceived route of HIV exposure (male-to-male sexual behaviour vs. other; as a proxy measure for MSM); and engagement in HIV-related peer support groups in the previous year (yes vs. no). Psychological distress was measured using the Kessler psychological distress scale (K6) [17]. This variable was dichotomized into a score of ≥ 13 vs. a score of < 13, using the recommended cut-off score to screen serious mental illness [17]. Histories of Illicit drug use included: ever used tryptamine-type derivatives (i.e., 5-MeO-DIPT or *Foxy* [N,N-diisopropyl-5-methoxytryptamine]), ever used cannabis, ever used methamphetamine/amphetamine, ever used MDMA, and ever used heroin/cocaine (all yes vs. no).

### Statistical analyses

First, socio-demographic and HV-related clinical characteristics were compared between males and females, using the Wilcoxon Rank Sum Test (for continuous variables) and the Pearson’s Chi-squared test (for categorical variables). Since the prevalence of drug use was extremely low among women (11.1% ever and 0% in the previous year), descriptive statistics were used to compare drug use patterns between males and females.

For bivariate and multivariate analyses of factors predicting past year quasi-legal psychoactive substance use among male participants, we used the survey logistic regression in the SAS software to account for possible clustering effects at the hospital level. A multivariate model was fit using an *a priori*-defined statistical protocol whereby all variables that were significantly associated with past year quasi-legal psychoactive substance use at the *p* < 0.05 level in the bivariate logistic regression analyses. In a sub-analysis, we examined the proportion of those who had never used any illicit drugs (i.e., tryptamine-type derivatives, cannabis, methamphetamine/amphetamine, MDMA, heroin or cocaine) among male participants who used quasi-legal psychoactive substance in the previous year. We also examined the proportion of those who used drugs for sexual activities in the previous year among the same aforementioned sub-sample. All *p*-values were two-sided. All statistical analyses were performed using SAS software version 9.3 (SAS, Cary, NC).

## Results

In total, 963 participants were eligible for the present study, including 45 (4.7%) females. Table 1 shows sample characteristics and drug use patterns, stratified by gender. As shown, the median age of the entire sample was 42 (interquartile range: 37–50) years. There was no significant gender-based difference in age. While female participants were more likely than males to be diagnosed with HIV before 2005, the majority of both males and females reported currently receiving ART. Annual income was significantly lower among women, compared to men. Among men, more than half reported a history of illicit drug use (including quasi-legal psychoactive substances), while only a handful of women did so. Among men, the most commonly used drug was quasi-legal psychoactive substances, followed by 5-MeO-DPT/*Foxy* and methamphetamine/amphetamine. Of note, the use of 5-MeO-DPT/*Foxy* in the previous year substantially dropped to 0.4%. Among women, the most commonly used drug was cannabis, followed by quasi-legal psychoactive substances and methamphetamine/amphetamine.

**Table 1:** Drug use patterns among HIV-positive male and female patients accessing HIV care in Japan (*n* = 963)

Characteristic	Total n (%)	Male	Female	*p* – value
		918 (95.3%)	45 (4.7%)	
Age (median, IQR)	42 (37–50)	42 (37–50)	41 (35–48)	0.449^1^
Calendar year of HIV diagnosis				<0.001^2^
1984–2005	337 (35.0%)	309 (33.7%)	28 (62.2%)	
2006–2009	302 (31.4%)	293 (31.9%)	9 (20.0%)	
2010–2013	324 (33.6%)	316 (34.4%)	8 (17.8%)	
Currently receiving ART	911 (94.6%)	870 (94.8%)	41 (91.1%)	0.289^3^
Annual income				0.044^4^
< 1 million Yen^a^	272 (28.3%)	253 (27.5%)	19 (42.2%)	
1–2.99 million Yen^a^	242 (25.1%)	231 (25.2%)	11 (24.4%)	
3–4.99 million Yen^a^	243 (25.2%)	231 (25.2%)	12 (26.7%)	
≥ 5 million Yen^a^	206 (21.4%)	203 (22.1%)	3 (6.7%)	
Quasi-legal psychoactive substance use^b^				
Ever	510 (53.0%)	508 (55.3%)	2 (4.4%)	
In the previous year	117 (12.2%)	117 (12.8%)	0 (0.0%)	
Tryptamine-type derivatives (i.e., 5-MeO-DPT or *Foxy*) use				
Ever	255 (26.5%)	255 (27.8%)	0 (0.0%)	
In the previous year	4 (0.4%)	4 (0.4%)	0 (0.0%)	
Cannabis use				
Ever	91 (9.5%)	88 (9.6%)	3 (6.7%)	
In the previous year	4 (0.4%)	4 (0.4%)	0 (0.0%)	
Methamphetamine/amphetamine use				
Ever	110 (11.4%)	109 (11.9%)	1 (2.2%)	
In the previous year	23 (2.4%)	23 (2.5%)	0 (0.0%)	
MDMA use				
Ever	58 (6.0%)	58 (6.3%)	0 (0.0%)	
In the previous year	4 (0.4%)	4 (0.4%)	0 (0.0%)	
Heroin use				
Ever	5 (0.5%)	5 (0.5%)	0 (0.0%)	
In the previous year	0 (0.0%)	0 (0.0%)	0 (0.0%)	
Cocaine use				
Ever	22 (2.3%)	22 (2.4%)	0 (0.0%)	
In the previous year	1 (0.1%)	1 (0.1%)	0 (0.0%)	
Use of any drugs listed above				
Ever	522 (54.2%)	517 (56.3%)	5 (11.1%)	
In the previous year	127 (13.2%)	127 (13.8%)	0 (0.0%)	

*IQR* interquartile range

^1^ *P*-value was derived from the Wilcoxon Rank Sum Test

^2^Chi-square value = 15.475, df = 2

^3^Chi-square value = 1.125, df = 1

^4^Chi-square value = 8.098, df = 3

^a^US$1.00 ≈ 100 Yen. “<1 million Yen” includes those without any income

^b^Includes variations of synthetic cannabinoids, synthetic cathinones, phenethylamine and thiophene derivatives, and amyl nitrites (e.g., *rush*, *poppers*). At the time of surveying, the possession or consumption of these substances was not prohibited

Table 2 shows the results of bivariate analyses of factors predicting past year quasi-legal psychoactive substance use among male participants. As shown, factors significantly associated with the outcome included: age; currently living alone; male-to-male sexual behaviour as a perceived route of HIV exposure; and all illicit drug use histories considered.

**Table 2:** Bivariate analyses of factors predicting past year quasi-legal psychoactive substance use among HIV-positive males accessing HIV care in Japan (*n* = 895)

Characteristic	Quasi-legal psychoactive substance use in the previous year		Odds Ratio (95% CI)	*p -* value
	Yes 115 (12.9%)	No 780 (87.1%)		
Age				
Median (IQR)	39 (33–44)	43 (37–52)		
Per year older			0.95 (0.93–0.97)	<0.001
Education				
< Secondary education	1 (0.9%)	21 (2.7%)	0.32 (0.07–1.53)	0.153
≥ Secondary education	114 (99.1%)	759 (97.3%)	ref	
Currently living alone				
Yes	61 (53.0%)	344 (44.1%)	1.43 (1.04–1.97)	0.028
No	54 (47.0%)	436 (55.9%)	ref	
Annual income				
< 1 million Yen^a^	28 (24.4%)	210 (26.9%)	0.76 (0.54–1.09)	0.444
1–2.99 million Yen^a^	29 (25.2%)	196 (25.1%)	0.85 (0.52–1.39)	0.995
3–4.99 million Yen^a^	28 (24.3%)	202 (25.9%)	0.80 (0.63–1.01)	0.518
≥ 5 million Yen^a^	30 (26.1%)	171 (22.1%)	ref	
ART use				
Currently on ART	106 (92.2%)	741 (95.0%)	0.62 (0.29–1.31)	0.210
Never on ART	9 (7.8%)	39 (5.0%)	ref	
Ever had AIDS				
Yes	32 (27.8%)	236 (30.3%)	0.89 (0.55–1.43)	0.625
No	83 (72.2%)	544 (69.7%)	ref	
Perceived route of HIV exposure				
Male-to-male sexual behaviour	111 (96.5%)	647 (82.9%)	5.70 (3.29–9.88)	<0.001
Other	4 (3.5%)	133 (17.1%)	ref	
Engagement in HIV-related peer support groups in the previous year				
Yes	9 (7.8%)	46 (5.9%)	1.36 (0.67–2.72)	0.394
No	106 (92.2%)	734 (94.1%)	ref	
Kessler psychological distress scale (K6)				
Score of ≥ 13	13 (11.3%)	99 (12.7%)	0.88 (0.48–1.61)	0.672
Score of < 13	102 (88.7%)	681 (87.3%)	ref	
Ever used tryptamine-type derivatives (5-MeO-DPT or *Foxy*)				
Yes	65 (56.5%)	187 (24.0%)	4.12 (2.90–5.86)	<0.001
No	50 (43.5%)	593 (76.0%)	ref	
Ever used cannabis				
Yes	25 (21.7%)	62 (8.0%)	3.22 (1.79–5.78)	<0.001
No	90 (78.3%)	718 (92.0%)	ref	
Ever used methamphetamine/amphetamine				
Yes	41 (35.7%)	68 (8.7%)	5.80 (3.79–8.88)	<0.001
No	74 (64.3%)	712 (91.3%)	ref	
Ever used MDMA				
Yes	19 (16.5%)	39 (5.0%)	3.76 (2.41–5.87)	<0.001
No	96 (83.5%)	741 (95.0%)	ref	
Ever used heroin or cocaine				
Yes	8 (7.0%)	15 (1.9%)	3.81 (1.62–9.01)	0.002
No	107 (93.0%)	765 (98.1%)	ref	

IQR: interquartile range. ART: antiretroviral therapy

^a^US$1.00 ≈ 100 Yen. “<1 million Yen” includes those without any income

Table 3 presents the results of the multivariate logistic regression analysis. As presented, age remained independently and negatively associated with past year quasi-legal psychoactive substance use. Factors that remained independently and positively associated with past year quasi-legal psychoactive substance use included: currently living alone; male-to-male sexual behaviour as a perceived route of HIV exposure; ever used 5-MeO-DPT/*Foxy*; and ever used methamphetamine/amphetamine.

**Table 3:** Multivariate logistic regression analysis of factors predicting past year quasi-legal psychoactive substance use among HIV-positive males accessing HIV care in Japan (*n* = 895)

Variable	Adjusted Odds Ratio	95% Confidence Interval	*p -* value
Age			
(per year older)	0.96	(0.93–0.99)	0.004
Living alone			
(yes vs. no)	1.66	(1.10–2.49)	0.015
Perceived route of HIV exposure			
(male-to-male sexual behaviour vs. other)	2.56	(1.48–4.43)	<0.001
Ever used tryptamine-type derivatives (5-MeO-DPT or *Foxy*)			
(yes vs. no)	2.42	(1.36–4.28)	0.003
Ever used cannabis			
(yes vs. no)	1.04	(0.51–2.12)	0.912
Ever used methamphetamine/amphetamine			
(yes vs. no)	3.59	(2.13–6.04)	<0.001
Ever used MDMA			
(yes vs. no)	0.90	(0.54–1.48)	0.667
Ever used heroin or cocaine			
(yes vs. no)	1.04	(0.56–1.96)	0.895

In the sub-analysis, among the 115 male participants who used quasi-legal psychoactive substances in the previous year, 38 (33.0%) reported having never used other illicit drugs. Sixty-nine (60.0%) individuals reported having used drugs for sexual activities in the previous year.

## Discussion

In our sample of PLHIV in Japan, illicit drug use was much more common among males than females. The most commonly used drugs among men were quasi-legal psychoactive substances. Over half of male participants reported having ever used quasi-legal psychoactive substances, and approximately 12% reported having used these substances in the previous year. The majority (97%) of those who used quasi-legal psychoactive substances in the previous year were presumably MSM. Approximately one-third of them had no history of using other illicit drugs. In the multivariate analysis, the lifetime use of 5-MeO-DPT/*Foxy* or methamphetamine/amphetamine, younger age, living alone, and male-to-male sexual behaviour as a perceived route of HIV exposure were independently and positively associated with recent quasi-legal psychoactive substance use among male PLHIV.

We found that male PLHIV who had ever used 5-MeO-DPT/*Foxy* were more likely to have used quasi-legal psychoactive substances in the previous year. While the cross-sectional nature of the study limits the assessment of temporal relationships, available data and literature suggest possible shifts in the patterns of drug use in this setting, from the use of fully banned to yet-to-be-fully-prohibited substances. As presented in Table 1, in our sample, 5-MeO-DPT/*Foxy* were second-ranked among the drugs that have ever been used in the lifetime (28%). However, past year use substantially dropped to <0.5%. Given that these tryptamine-type derivatives became fully prohibited under the Narcotics and Psychotropics Control Law in 2005, our findings suggest that “substance displacement” might have occurred in this setting [18] whereby those who had used these illegal stimulants may have shifted to other quasi-legal psychoactive substances with similar psychoactive effects as these psychoactive substances became easily available. This is also congruent with previous forensic laboratory reports suggesting that stricter controls of certain substances have met with the emergence of new analogues of controlled substances in Japan [16].

Likewise, the independent association between lifetime use of methamphetamine/amphetamine and recent use of quasi-legal psychoactive substances could also be interpreted as switching from the former to the latter. However, a recent qualitative study reported that quasi-legal psychoactive substance use might subsequently lead to the use of methamphetamine/amphetamine among some PLHIV in Japan [19]. In light of this previous study finding, the observed association may also suggest that some of those who used quasi-legal psychoactive substances in the previous year subsequently used methamphetamine/amphetamine. Future research should determine the potential role of quasi-legal psychoactive substance use in the initiation of methamphetamine/amphetamine use in this setting.

We also found that those who were younger and those who were likely MSM were more likely to report recent use of quasi-legal psychoactive substances. This is consistent with a previous study conducted in Tokyo [12]. According to a recent qualitative investigation of patterns of illicit drug use among HIV-positive MSM in Japan, illicit drugs (including quasi-legal psychoactive substances) were used for several purposes in this population, including as a strategy to cope with depression and sexual minority stress, and as a sexual stimulant [20]. Regardless of the purposes, illicit drug use often resulted in increasing sexual HIV risk behaviour [20]. Consistent with these qualitative research findings, in our study, two-thirds of the past year quasi-legal psychoactive substance users reported having used drugs for sexual purposes. Previous literature also indicated high rates of sexual stimulant use among HIV-positive MSM in other countries [21]. Ongoing quasi-legal psychoactive substance use and the potentially associated risky sexual behaviour among HIV-positive MSM are concerning. They warrant greater efforts to address factors that lead to illicit drug use and to reduce drug-related harms in this setting.

Internationally, identifying effective regulations of new psychoactive substances is a major topic of debate [22, 23]. In April 2014, the Japanese government penalized the personal possession and consumption of all Designated Substances [24]. This legislation change in Japan, which outright banned more than 1,300 Designated Substances [24], could be viewed as a bold attempt to contain the illicit psychoactive substance market through law enforcement. Some commentators raised a concern regarding the overreliance on such law enforcement-based approaches [25]. They claimed that as new psychoactive substances are moving targets, such approaches may potentially increase the drug-related harm among those who continue to use those controlled substances [25]. Our findings suggesting the possible shift from the use of banned to unregulated substances reinforce the concern. Given that new unregulated substances may pose increased or different kinds of harm, the findings indicate a need to address various social and structural factors that predispose vulnerable populations to illicit drug use and the associated harm.

Our study has several limitations. First, as the study sample was not randomly selected, our findings may not be generalizable to PLHIV in Japan or elsewhere. Second, the self-reported data may be affected by reporting biases, including recall bias and socially desirable responding. However, we note that the anonymous nature of the questionnaire may have helped reduce some of the biases. Third, as with all observational studies, the associations between the explanatory variables and outcome assessed may be under the influence of unmeasured confounding factors. However, we sought to address this bias with multivariate adjustment of the key demographic and behavioural predictors of quasi-legal psychoactive substance use.

## Conclusion

In sum, illicit drug use was much more common among male patients in HIV care in Japan, compared to their female counterparts. Among male participants, quasi-legal psychoactive substances were the most commonly used drugs. We also found a substantial decline in recent use of 5-MeO-DPT/*Foxy*. Individuals who had ever used these tryptamine derivatives or methamphetamine/amphetamine were more likely to report recent quasi-legal psychoactive substance use. These findings suggest a potential shift in drug use patterns from regulated to unregulated substances among this population. Given that a new regulation instituted in April 2014 fully banned presumably most of the quasi-legal psychoactive substances considered in this study, further research is warranted to examine changing drug patterns and implications for HIV care in this setting.

## Abbreviations

AIDS: Acquired immune deficiency syndrome
HIV: Human immunodeficiency virus
MSM: Men who have sex with men
PLHIV: People living with HIV/AIDS

## References

[1] Joint United Nations Programme on HIV/AIDS (2016). AIDS by the numbers.

[2] Hser Y-I, Liang D, Lan Y-C, Vicknasingam BK, Chakrabarti A (2016). Drug abuse, HIV, and HCV in Asian countries. J Neuroimmune Pharmacol.

[3] Degenhardt L, Mathers B, Guarinieri M, Panda S, Phillips B, Strathdee SA (2010). Meth/amphetamine use and associated HIV: implications for global policy and public health. Int J Drug Policy.

[4] Shoptaw S, Montgomery B, Williams CT, El-Bassel N, Aramrattana A, Metsch L (2013). Not just the needle: the state of HIV-prevention science among substance users and future directions. J Acquir Immune Defic Syndr.

[5] Shoptaw S, Reback CJ (2007). Methamphetamine use and infectious disease-related behaviors in men who have sex with men: implications for interventions. Addiction.

[6] Beyrer C, Baral SD, van Griensven F, Goodreau SM, Chariyalertsak S, Wirtz AL (2012). Global epidemiology of HIV infection in men who have sex with men. Lancet.

[7] Rajasingham R, Mimiaga MJ, White JM, Pinkston MM, Baden RP, Mitty JA (2012). A systematic review of behavioral and treatment outcome studies among HIV-infected men who have sex with men who abuse crystal methamphetamine. AIDS Patient Care STDs.

[8] Global SMART Programme (2015). The challenge of synthetic drugs in East and South-East Asia and Oceania: trends and patterns of amphetamine-type stimulants and new psychoactive substances.

[9] Wada K (2011). The history and current state of drug abuse in Japan. Ann N Y Acad Sci.

[10] Suguimoto SP, Techasrivichien T, Musumari PM, El-saaidi C, Lukhele BW, Ono-Kihara M (2014). Changing patterns of HIV epidemic in 30 years in East Asia. Curr HIV/AIDS Rep.

[11] Wada K, Funada M, Shimane T (2013). Current status of substance abuse and HIV infection in Japan. J Food Drug Anal.

[12] Nishijima T, Gatanaga H, Komatsu H, Takano M, Ogane M, Ikeda K (2013). High prevalence of illicit drug use in men who have sex with men with HIV-1 infection in Japan. PLoS One.

[13] Nishijima T, Gatanaga H, Komatsu H, Takano M, Ogane M, Ikeda K (2013). Illicit drug use is a significant risk factor for loss to follow up in patients with HIV-1 infection at a large urban HIV clinic in Tokyo. Paraskevis D, editor. PLoS One.

[14] Nishijima T, Shimbo T, Komatsu H, Hamada Y, Gatanaga H, Oka S (2014). Incidence and risk factors for incident hepatitis C infection among men who have sex with men with HIV-1 infection in a large urban HIV clinic in Tokyo. J Acquir Immune Defic Syndr.

[15] Kikura-Hanajiri R, Uchiyama N, Kawamura M, Ogata J, Goda Y (2013). Prevalence of new designer drugs and their legal status in Japan. Yakugaku Zasshi.

[16] Kikura-Hanajiri R, Kawamura NUM, Goda Y (2014). Changes in the prevalence of new psychoactive substances before and after the introduction of the generic scheduling of synthetic cannabinoids in Japan. Drug Test Anal.

[17] Kessler RC, Barker PR, Colpe LJ, Epstein JF, Gfroerer JC, Hiripi E (2003). Screening for serious mental illness in the general population. Arch Gen Psychiatry.

[18] United Nations Office on Drugs and Crime (2008). 2008 World drug report.

[19] Ikushima Y, Nosaka Y, Okamoto G, Yamagushi M, Nakayama M, Otsuki T (2013). Associations between HIV and drug use among HIV-positive people who have a history of drug use: a qualitative study.

[20] Ikushima Y, Nosaka Y, Okamoto G, Yamagushi M, Nakayama M, Otsuki T (2014). Associations between HIV and drug use among people who have a history of drug use: a qualitative study.

[21] Wei C, Guadamuz TE, Lim SH, Huang Y, Koe S (2012). Patterns and levels of illicit drug use among men who have sex with men in Asia. Drug Alcohol Depend.

[22] Hughes B, Griffiths P (2014). Regulatory approaches to new psychoactive substances (NPS) in the European Union. Addiction.

[23] Wilkins C (2014). A critical first assessment of the new pre-market approval regime for new psychoactive substances (NPS) in New Zealand. Addiction.

[24] Ministry of Health, Labour and Welfare (Japan) (2014). Possession, use, etc. of designated substances will be prohibited, beginning on April 1, 2014.

[25] Wodak AD (2014). New psychoactive substances: reducing the harm caused by untested drugs and an unregulated market. Med J Aust.

